#  Ethnic Variations in Perception of Human Papillomavirus and its Vaccination among Young Women in Nepal .

**DOI:** 10.3126/nje.v7i1.17757

**Published:** 2017-03-31

**Authors:** Brijesh Sathian, M G Ramesh Babu, Edwin R. van Teijlingen, Indrajit Banerjee, Bedanta Roy, Supram Hosuru Subramanya, Elayedath Rajesh, Suresh Devkota

**Affiliations:** 1 Assistant Professor, Department of Community Medicine, Manipal College of Medical Sciences Pokhara, Nepal; 2 Senior Lecturer, Department of Physiology, Melaka Manipal Medical College Manipal University, India; 3 Professor, School of Health & Social Care, Bournemouth University Bournemouth, UK.; 4 Associate Professor, Department of Pharmacology, Chitwan Medical College and Teaching Hospital Chitwan, Nepal; 5 Assistant Professor, Department of Physiology, Manipal College of Medical Sciences Pokhara, Nepal.; 6 Assistant Professor, Department of Microbiology, Manipal College of Medical Sciences Pokhara, Nepal.; 7 Assistant Professor, School of Behavioural Sciences, Mahatma Gandhi University India.; 8 Lecturer, Department of Community Medicine, Manipal College of Medical Sciences Pokhara, Nepal.

**Keywords:** Human papillomavirus, HPV vaccination, HPV awareness, cervical cancer, Youth.

## Abstract

**Background:**

The Human Papillomavirus (HPV) is strongly associated with cervical and other cancers. In women, cervical cancer is the third most common cancer. HPV infection can be largely prevented through vaccination of (adolescent) girls. At the same time, Nepal is a low-income country experiencing a cultural change in attitudes towards sex and sexual behaviour. However, in the adolescent population knowledge about HPV, factors associated with an increased risk of HPV and the existence of the vaccination is often low.

**Materials and Methods:**

This was a cross-sectional study with female students enrolled in health and non-health science courses in Pokhara, Nepal. The questionnaire included demographic details, knowledge and attitude questions related to HPV, associated risk behaviour and its vaccination. Descriptive statistics, including Chi-Square test, were used to identify statistically significant relationships. Ethical approval was granted by the relevant authority in Nepal.

**Results:**

Hindu religion (75.0 %; 95% CI: 70.9, 78.6) and Newari caste (75.5%; CI: 61.1, 86.7) were more aware about HPV, HPV vaccination. Hindus religion (55.6%; 95% CI: 51.2, 60.0) and Dalit caste (61.6%, 95% CI: 53.3, 69.4) more willing to be vaccinated than other religions and other castes, respectively. Not unsurprisingly, students on health-related courses had a greater awareness of HPV, HPV vaccination and were more willing to be vaccinated than students on other courses. Similar patterns of association arose for knowledge related to those sexually active at an early age; HPV risk and multiple sex partners; and fact that condoms cannot fully prevent the transmission of HPV.

**Conclusion:**

Knowledge about the link between HPV and (a) early sexual initiation, (b) having multiple sexual partners, and (c) the limited protection of condoms and other birth control measures was poor in our study compared to similar research conducted in other parts of the world. One key implication is the need for education campaigns in Nepal to educate young women and their parents about HPV, its risk factors and the benefits of vaccination. .

## Introduction

Genital Human papillomavirus (HPV) infection is the most common sexually transmitted infection (STI) throughout the world which causes 99.7% of cervical cancer, 90% of anal cancer, 40% of vulvar, vaginal, or penile cancers, and 12% of oral and pharyngeal cancers [ 1, 2 ]. It is commonly seen in women who had a higher number of sexual partners or had sex with men, who had sex with multiple partners [ 3, 5 ]. It is estimated that more than 80% of sexually active women will have genital HPV by the time they reach the age of 50 years [ 1 ]. Although most infections are transient and asymptomatic, persistent infection with a high-risk type of HPV can lead to precancerous lesion and progress to cancer [ 6, 7 ]. The most common cancer in women is cervical cancer which can be lethal once it reaches the invasive stages. However, out of all the female genital tract cancers, it is the only preventable cancer if detected early enough [ 1, 8 ]. A data-based study in 2008 on cervical cancer has indicated that there were 275,000 deaths out of an estimated 530,000 cervical cancer cases worldwide. 85% of the global burden due to cervical cancer falls to Low-Income Countries with overall 52% mortality incidence ratio and cervical cancer is responsible for about 88% of death which occur in developing countries. This suggests a lack of effective public health control measures and lack of infrastructure in many developing countries [ 5, 9 ]. In a low-income country like Nepal, where traditional sexual norms are changing, more young people are at risk of developing STIs including HPV than in the past. The risk of developing HPV-related disease has become endemic in regions that support child marriages, polygamy, high parity, and long-term oral contraceptive use [ 5, 7 ]. Cervical cancer is one of the most commonly reported cancer among women in Nepal and has higher incidence rate (14.9) than other HPV-related cancers [ 10, 12 ]. The age-standardized rate (ASR) of cervical cancer for women in Nepal was found to be 19.0 for the year 2014. Every year 1367 die out of 2332 women who are diagnosed with cervical cancer in Nepal. [ 12 ]. Fortunately, there is an important secondary preventive measure for cervical cancer such as effective Population-based screening, Pap smear testing, HPV DNA testing, visual inspection with acetic acid and HPV vaccine, that leads to a high cure rate among cervical cancer patients [ 1, 5, 13 ].

Vaccination will be most effective if it is given before the onset of the sexual activity and will be useful who are already sexually active [ 14 ]. A 9-valent-HPV Vaccine, a recent investigation by Joura et al, can prevent infection and disease related to HPV (HPV Types; 6, 11, 16, 18, 31, 33, 45, 52, and 58) [ 15 ]. Singh et al. had conducted an HPV vaccination programme in school girls in Nepal with help of Australian Cervical Cancer Foundation and suggested that high cost and low public awareness were the key barriers to successful implementation of a vaccination programme in Nepal [ 16, 13 ]. Though Nepal is one of the "countries eligible for GAVI Alliance support " for vaccination [ 17 ], since the incidence rate of cervical cancer is high, vaccination programme will require major donor funding until mature, affordable prices are achieved [ 2 ]. Apart from high cost, religious beliefs, practicing cast system, parent's acceptance, lack of permission from husband and lack of public awareness also influence on making decision for vaccination and prevents women from participating in screening programme. Though vaccination is a most successful public health approach in protecting and controlling HPV infection [ 14 ], for successful implementation of the vaccine, it is necessary to bring awareness regarding HPV, cervical cancer, and the benefits of HPV vaccination among young women. Because it will be difficult for women to undergo screening test and getting vaccinated in the absence of symptoms [ 10 ]. Public health programmes may help to overcome such difficulties but such programmes are only going to succeed if the level of HPV awareness among women is high. Since HPV poses a greater threat to women's health than men's, research has focused primarily on women's awareness and knowledge of HPV. Various studies have evaluated women's awareness and knowledge levels of cervical cancer and HPV [ 18, 23 ]. Although a number of studies worldwide found women's knowledge to be poor, there appears to be strong support for HPV vaccination [ 1, 24, 26 ]. Therefore, establishing the knowledge and awareness about HPV and its vaccination in women and identifying sub-groups of women in the target age group for HPV vaccination, who are having lower levels of knowledge, is crucial before any screening test and vaccination programme. The objective of our study was to establish the knowledge of HPV and its vaccination among female students studying in Pokhara, Nepal.

## Methodology

### Study design and the participants:

This cross-sectional study was carried out during July-December, 2013 in a community setting with female undergraduate students of Pokhara sub metropolitan city, Western Nepal. Pokhara has 28 Wards, 82870 House Hold, and population as 152651 Male, 161190 Female, 313841 Total. A cross-sectional survey can provide key information on associations and risk factors [ 27 ].

### Data collection:

The questionnaires were administered in face-to-face interviews by trained researchers. Data were collected from female students who are educated in English in their colleges in Pokhara, Nepal. This study used a convenience sample.

### Questionnaire:

An extensive review of the literature, combined with exploratory research and qualitative piloting [ 28 ] contributed to the development and refinement of a structured questionnaire in English. The questionnaire was validated using reliability analysis resulting in a high Cronbach's alpha score of 0.801. Therefore, the questionnaire was not changed after piloting. Those who completed the pilot were also included in the study. We used the information from the pilot study for calculating the required sample size. The questionnaire included questions on HPV awareness as well as knowledge of risk factors associated with HPV transmission. The latter included questions such as whether the sexual activity at an early age can cause HPV-related cervix cancer (risk), sexual contact with multiple partners (transmission), and whether condoms offered protection (measures to control). The items were not grouped according to the type of question so that the individual influence of each variable could be examined.

### Inclusion criteria:

All the female students who are educated in English in their colleges were included in the study.

### Exclusion criteria:

Those who refused to consent were excluded from the study.

### Sample size calculation:

A total of 700 female under graduate students were invited to the questionnaire study. The sample size calculation suggested that for a 95% confidence interval and significance level á = 5%, P = 0.525, Q = 0.475, allowable error = 8%, whereby P = awareness of HPV in Nepal [ 29, 30 ], the required sample size was 543.

### Outcome Variable:

The respondents’ answers to the questionnaire were coded in a yes/no response format. Dependent variables were awareness about HPV infection, HPV vaccination, HPV-related cervix cancer.

### Explanatory variables:

The demographic factors such as age, religion, and ethnicity were taken as the independent variables.

### Ethical committee approval:

Ethics approval was obtained prior from the Institutional Research and Ethics Committee of Manipal College of Medical Sciences, Pokhara, Nepal (affiliated to Kathmandu University) which is authorized by Nepal Health Research Council (NHRC). Written consent was obtained from the participants. The researchers described the purpose and process of study to the respondents and emphasized confidentiality and anonymity. It was made very clear to the participants that they had the freedom of not participating in the study. All the concerns raised by the participants were addressed. Our research was conducted in accordance with the Declaration of Helsinki [ 32].

### Data management and statistical analysis:

The data collected were analyzed using EPI-Info 3.5.1 Windows Version. Descriptive statistics, testing of hypothesis with Chi-Square test for finding the relationship between two variables and 95% confidence intervals (95% CI) were used to interpret the data [ 31].

## Results

### Sample Characteristics

The interview was conducted in 700 female students and 685 students were completed the interview. Totally 15 students were dropped out from the study due to poor response. Since overall response rate was high (97.86%) and the numbers students (n=685) after dropouts (n=15) were not less than the calculated sample size (n=578), removing 15 students from the study did not have any impact on our result. The average age of the students was 18.5 (Mean ± SD; 18.56 ± 0.831) with the range of 18 to 21 years of age. Most of the students were belong to Hindu religion [507 (74%)] and Chettri caste [180 (26.1%)]. The number of non-health science students [362 (52.8%)] were slightly more than health science students [323 (47.2%)]. All other socio-demographic details of the students were shown in  Table 1. On considering knowledge of the students about HPV, HPV vaccine, and sexual behavior, most of the students (71.7 %) knew about HPV, but only 51.8% of the students knew about the HPV vaccine and they were willing to be vaccinated. Over one-third of the students knew risk (37.5%), transmission (44.1%) and measures to control of HPV (40.4%).

### Knowledge and attitudes towards HPV by Religion, Caste, and Education

Table 2 shows that more Hindu students were aware of HPV (75.0%; 95% CI: 70.9, 78.6), HPV vaccination and also willing to be vaccinated (55.6%; 95% CI: 51.2, 60.0) than students of other religions. A significant number of Muslim students (70.0%; 95% CI: 50.6, 85.3) were not willing to be vaccinated thought they were aware of HPV. On considering the knowledge of students related to risk, transmission, and measures to control HPV, many students had selected the option “No” in the questionnaire. Muslim students had less knowledge related to risk (73.3%; 95% CI: 54.1, 87.7), transmission (80.0%; 95% CI: 61.4, 92.3) and measures to control (73.3%; 95% CI: 54.1, 87.7) HPV.

Table 3 suggests that the majority of students (P=0.01) of different castes were aware of HPV, but only Brahmin (52%; 95% CI: 43.3, 61.5), Newari (61.2%; 95% CI: 46.2, 74.8), and Dalit (61.6%; 95% CI: 53.3, 69.4) castes students were showed their willingness for vaccination against HPV. Whereas only 50% of Chettri caste students were willing to be vaccinated. On comparing the knowledge related to risk, transmission, and measures to control HPV among different caste students, most of them did not have the knowledge about it. But slightly more number of students in Newari (53.1%; 95% CI: 38.3, 67.5) and Dalit (51.7%; 95% CI: 43.4, 59.9) castes were known about the transmission of HPV through multiple sex partners.

On comparing knowledge and attitude towards the HPV between health science and non- health science students, a significant number of health science students (98.8%; 95% CI: 96.6, 99.6; p < 0.001] were aware of HPV, its vaccination and willing to be vaccinated. Almost identical patterns occurred for knowledge related to risk, transmission, and measures to control HPV (p<0.001) which is shown in Table 4.

## Discussion:

### Education

Women's participation in all sectors of development has increased tremendously worldwide. Although Nepal has the most dismal female literacy rate in the SAARC (South Asia association for Regional Cooperation) [ 33 ], the percentage of school going children is increasing trend in recent years [ 34 ]. The increasing formal education rates of adolescent girls have a positive effect on their overall understanding of health and disease. The widespread awareness programme nowadays has certainly brought significant changes in adolescent girl's attitude towards diseases like HPV [ 35, 41 ]. Effect of such awareness programmes was reflected in the present study which was carried out with female undergraduate students studying in Pokhara valley, Nepal. The result of the present study reveals that most of the students were aware HPV and Pap smear test (71.2%) which is shown in Table 4. Similar findings have been reported in the Middle East on levels of knowledge and awareness of the Pap smear test [ 42, 43 ], whilst women in Brazil had poor knowledge about HPV (70.9%) and also the Pap smear test (53.0%) [ 44 ]. The result of the present study is contrary to the study by Johnson et al. who reported that only 15.4% women in a village in Nepal heard about HPV [ 45 ]. It indicates the importance of women education and HPV awareness programmes in Nepal should reach village level for a better understanding of public health related diseases. When comparing health science students with non-health science students in the present study, we observed that health science students were more aware (98.8%) of HPV than non-health science students (47.5%). On considering knowledge about vaccine and willingness for vaccination, it is surprising that only 51.8% knew about HPV vaccine and were willing to be vaccinated. Only health science students (98.8%) showed their willingness for vaccination when compared with non-health science students (9.9%). It is discordant with the existing literature on HPV vaccine knowledge and acceptability in both high and low-income settings, which is that while levels of knowledge are generally low, individuals are still willing to receive vaccination against HPV [ 46, 52 ]. Similarly on considering knowledge related to the risk, transmission, and measures to control of HPV, again health science students significantly differ (P < 0.0001) from non-health science students. It shows that health science students have some benefit of health science teaching on an understanding of health related diseases like HPV.

### Religion

Since Hinduism is the major religion of Nepal (86.51%), most of the students in this study were Hindus (74%). Most of them were aware of HPV and Pap smear test (75%) and were showed their willingness for HPV vaccination (55.6%) when compared with other religious students. In contrary to the present study, Joy et al. had reported in his study that more Christians were aware of HPV than other religions, which was conducted in 2011 in medical and allied-health professional schools in Pokhara [ 29 ]. In the present study, students belong to Christians, Muslims and Buddhist religion were aware of HPV, but not willing to get a HPV vaccination, particularly Muslim students (p<0.001). Religious beliefs play an important role in determining for vaccination. As reported by Guimond, in the Muslim culture, modesty is associated with religious beliefs and practices. Lack of sensitivity to modesty is a barrier for Muslim women to obtain cervical cancer screening and prevention [ 53 ]. However, it is difficult to decide the influence of religious beliefs on making a decision about vaccination by just interviewing students in the present study. A study by Pelucchi et al. reported that more mothers than fathers were aware of HPV infection and favorable towards vaccinating their children against HPV [ 54 ]. A recent study in adolescent girls among a mixed population of Hindu, Muslim and Christian parents in India by Madhivanan et al. had reported that 71% of parents were showed their willingness to accept HPV vaccine for their daughters [ 55 ]. However, as cited by Marlow et al." parents with strong religious or cultural views were less likely to accept HPV vaccination " [ 56 ]. Therefore population-based studies, including student's parents, are necessary to know about parents and students willingness for HPV vaccination. Students from all other religions had poor knowledge related to the link between HPV and (a) risk; (b) transmission; and (c) measures to control, when compared to some studies from other parts of the world [ 35, 57, 61 ].

### Caste

In the present study, on considering knowledge and attitudes of students towards HPV against caste system, students from all different castes were aware of HPV and Pap smear test. But only 52% of Brahmin, 61.2% of Newari, 61.1% of Dalit and 50% of Chettri caste students showed their willingness for vaccination. While considering knowledge about the link between HPV and (a) risk; (b) transmission; and (c) measures to control, most of them had poor knowledge about it. Though Dalit girls are still getting less access to education, health care, and other opportunities than other higher castes groups in Nepal, implementation of scholarship programme made Dalit students to access education in schools [ 34 ]. This is very well reflected in our study with more number of Dalit students (22%) next to Chettri (26.3%) students studying in medical and non-medical schools. Considering their knowledge about HPV among Dalit students, a higher number of students were aware of HPV (82.1%), willing to be vaccinated (61.6%) and slightly more students knew about the transmission of HPV through multiple sex partners (51.7%). Religious beliefs and attitude towards health and diseases have been changing nowadays. Sex is no longer a social taboo among many ethnic groups [ 62 ]. But this promiscuity had led more chances of spread of sexually transmitted diseases like HPV especially among Dalit caste [ 34 ]. A community-based health education programme should be implemented in Nepal to increase awareness and risk factors associated with HPV. Our study supports previous work showing that it is very important to educate people in Nepal about the transmission of HPV and the associated risk factors, [ 63, 64 ] and the benefits of HPV vaccine. Educating people about HPV and its vaccination will elevate public trust, which is a critical component of successful implementation of widespread vaccine coverage. There are several examples which proved that awareness programme about HPV and its vaccination in countries like Japan, India and Rwanda [ 39, 41, 65, 67 ] have increased public trust in HPV vaccine.

## Conclusion

Our study outcome highlights the need for culturally salient HPV education among the Nepalese population. Education, religious beliefs, and caste can significantly influence in gaining knowledge about HPV which is reflected in the present study. A significant difference in knowledge and attitude towards HPV vaccination among different religious and ethnic groups are the interesting findings of this study. This study may give an insight to the Ministry of Health & Population and (international) non-governmental organizations working on the implementation of HPV vaccination in Nepal. Though women education and their participation in all sectors are increasing in Nepal, the social customs can place women at an increased risk of contracting human papillomavirus (HPV) [ 5 ]. Strong recommendations from health workers could influence the women in deciding to get vaccinated. The professionals should also be educated and trained about HPV vaccination, and its relation with cervical cancer to increase awareness and knowledge about HPV to women [ 68 ]. However detailed studies including both male and female students and their parents in different parts of the Nepal are essential to bring HPV awareness, implement of a vaccine against HPV and to eliminate the risk of HPV.

### Strength and limitations of the study

Our study is one of the first of its kind in Nepal with large sample size, which is allowed us for comparisons between important social characteristics such as religion and caste, is the key strength of this study. It identifies the barriers to HPV vaccination with respect to religious beliefs and practicing caste system. But parents are not included in this study who are the deciding authority of students for vaccination, which is one of the limitations in this study. Though this study was conducted in Pokhara, Nepal, which might not reflect the levels of knowledge about HPV in other parts of the Nepal, this study may be helpful and taken as reference for studies and HPV vaccination programme in future. One of the questions appeared as double question ‘Do you know about HPV vaccine and are you willing to be vaccinated?’ We can never know about the people who would have said ‘yes’ to having heard about HPV vaccine and ‘no’ about being willing to be vaccinated and vice versa.

### Future scope of the study:

A multi-stage government funded large sample national based study should be carried out to find out the actual scenario in Nepal.

### What is already known on this topic:

Few studies have been conducted in different parts of the Nepal showing that knowledge and awareness of HPV, cervical cancer and HPV vaccine among the Nepali women.

### What this study adds:

This study reveals the association between religion and caste on the one hand and on the other knowledge and awareness of HPV, cervical cancer and HPV vaccine among the Nepali women.

## Figures and Tables

**Table:** Table 1: Socio-demographic characteristics of the respondents

Variables	n (%)
Religion	
Hindu	507 (74.0)
Muslim	30 (4.4)
Christian	32 (4.7)
Buddhist	116 (16.9)
**Caste**	
Brahmin	124 (18.1)
Chettri	180 (26.3)
Newar	49 (7.2)
Gurung	77 (11.2)
Dalit	151 (22.0)
Magar, Pun, Lama	39 (5.7)
Other	65 (9.5)
**Education**	
Non-Medical	362 (52.8)
Medical/ Health Science	323 (47.2)
**Total**	685

**Table:** Table 2: Knowledge and attitudes towards HPV by religion

		Religion				
		n [(%(95%CI)]				
Questionnaire Items		Hindu	Muslim	Christian	Buddhist	p value
Do you know about HPV vaccine and are you willing to be vaccinated?	No	225[44.4(40.0, 48.8)]	21[70.0(50.6, 85.3)]	17[53.1(34.7, 70.9)]	67[57.8(48.2, 66.9)]	0.004†
	Yes	282[55.6(51.2, 60.0)]	9[30.0(14.7, 49.4)]	15[46.9(29.1, 65.3)]	49[42.2(33.1, 51.8]	
Are you aware of human papillomavirus (HPV) and pap smear test?	No	127[25.0(21.4, 29.1)]	12[40.0(22.7, 59.4)]	10[31.3(16.1, 50.0)]	45[38.3(29.9, 48.3)]	0.001†
	Yes	380 [75.0(70.9,78.6)]	18[60.0(40.6, 77.3)]	22[68.8(50.0, 83.9)]	71[61.2(51.7, 70.1)]	
Do you know being sexually active at an early age (before age 16) increases the chance of HPV-related cervical cancer?	No	310[61.1(56.7, 65.4)]	22[73.3(54.1, 87.7)]	21[65.6(46.8, 81.4)]	75[64.7(55.2, 73.3)]	0.520*
	Yes	197[38.9(34.6, 43.3)]	8[26.7(12.3, 45.9)]	11[34.4(18.6, 53.2)]	41[35.3(26.7, 44.8)]	
Do you know HPV can be transmitted through multiple sex partners?	No	266[52.5(48.0, 56.9)]	24[80.0(61.4, 92.3)]	19[59.4(40.6, 76.3)]	74[63.8 (54.4,72.5)]	0.006†
	Yes	241[47.5(43.1, 52.0)]	6[20.0(7.7, 38.6)]	13[40.6(23.7, 59.4)]	42[36.2(27.5, 45.6)]	
Do you know using of condom/other birth control measures cannot prevent fully the transmission of HPV?	No	292[57.6(53.2, 61.9)]	22[73.3(54.1, 87.7)]	19[59.4(40.6, 76.3)]	75[64.7(55.2, 73.3)]	0.219*
	Yes	215[42.4(38.1, 46.8)]	8[26.7(12.3, 45.9)]	13[40.69(23.7,59.4)]	41[35.3 (26.7,44.8)]	
* Not Statistically Significant [p-value ≥ 0.05]						
† Statistically Significant [p-value < 0.05]						

**Table:** Table 3 Knowledge and attitudes towards HPV by caste

Caste n [(%(95%CI)]									
Questionnaire Items		Brahmin	Chettri	Newar	Gurung	Dalit	Magar, Pun, Lama	Other	p value	
Do you know about HPV vaccine and are you willing to be vaccinated?	No	59 [47.6(38.5, 56.7)]	90[50.0(42.5, 57.5)]	19[38.8(25.2, 53.8)]	42[54.5(42.8,65.9)]	58[38.4(30.6, 46.7)]	25[64.1(47.2, 78.8)]	37[56.9(44.0, 69.2)]	0.021†	
	Yes	65[52(43.3, 61.5)]	90[50(42.5, 57.5)]	30[61.2(46.2, 74.8)]	35[45.5(42.8,65.9)]	93[61.6(53.3, 69.4)]	14[35.9(21.2, 52.8)]	28[43.1(30.8, 56.0)]		
Are you aware of human papillomavirus (HPV) and pap smear test?	No	33[26.6(19.1, 35.3)]	55[30.6(23.9, 37.8)]	12[24.5(13.3, 38.9)]	29[37.7(26.9,49.4)]	27[17.9(12.1, 22.7)]	16[41.0(25.6, 57.9)]	22[33.8(22.6, 46.6)]	0.011†	
	Yes	91[73.4(64.7, 80.9)]	125[69.4(62.2, 76.1)]	37[75.5(61.1, 86.7)]	48[62.3(50.6,73.1)]	124[82.1(75.1, 87.5)]	23[59.0(42.1, 74.4)]	43[66.2 (53.4, 77.4)]		
Do you know being sexually active at an early age (before age 16) increases the chance of HPV-related cervical cancer?	No	75[60.5(51.3, 69.1)]	108[60.0(52.4, 67.2)]	28[57.1(42.2, 71.2)]	49[63.6(51.9,74.3)]	95[62.9(54.7, 70.6)]	26[66.7(49.8, 80.9)]	47[72.3(72.3, 82.7)]	0.636*	
	Yes	49[39.5(30.9, 48.7)]	72[40.0(32.8, 47.6)]	21[42.9(28.8, 57.8)]	28[36.4(25.7,48.1)]	56[37.1 (29.4, 45.3)]	13[33.3(19.1, 50.2)]	18[27.7(17.3, 40.2)]		
Do you know HPV can be transmitted through multiple sex partners?	No	71[57.3(48.1, 66.1)]	97[53.9(46.3, 61.3)]	23[46.9(32.5, 61.7)]	45[58.4(46.6,69.6)]	73[48.3(40.1, 56.6)]	29[74.4(57.9, 87.0)]	45[69.2(56.6, 80.1)]	0.015†	
	Yes	53[42.7(33.9, 51.9)]	83[46.1(38.7, 53.7)]	26[53.1(38.3, 67.5)]	32[41.6(30.4,53.4)]	78[51.7 (43.4, 59.9)]	10[25.6(13.0, 42.1)]	20[30.8 (19.9, 43.4)]		
Do you know using of condom/other birth control measures cannot prevent fully the transmission of HPV?	No	77[62.1(52.9, 70.7)]	105[58.3(50.8, 65.6)]	27[55.1(40.2, 69.3)]	46[59.7(47.9,70.8)]	81[53.6(45.4, 61.8)]	29[74.4(57.9, 87.0)]	43[66.2(53.4, 77.4)]	0.256*	
	Yes	47[37.9(29.3, 47.1)]	75[41.7(34.4, 49.2)]	22[44.9(30.7, 59.8)]	31[40.3(29.2,52.1)]	70[46.4(38.2, 54.6)]	10[25.6(13.0, 42.1)]	22[33.8(22.6, 46.6)]		
* Not Statistically Significant [p-value ≥ 0.05]									
† Statistically Significant [p-value <0.05]									

**Table:** Table 4: Knowledge and attitudes towards HPV by type of education

Education (type) n [(%(95%CI)]				
Questionnaire Items		Non-Medical / Health	Medical/ Health Science	p Value
Do you know about HPV vaccine and are you willing to be vaccinated?	No	326[90.1(86.5, 92.9)]	4[1.2(0.4, 3.4)]	p < 0.0001†
	Yes	36[9.9(7.2, 13.6)]	319[98.8(96.6, 99.6)]	
Are you aware of human papillomavirus (HPV) and pap smear test?	No	190[52.5(47.2, 57.7)]	4[1.2(0.4, 3.4)]	p < 0.0001†
	Yes	172[47.5(42.3,52.8)]	319[98.8(96.6, 99.6)]	
Do you know being sexually active at an early age (before age 16) increases the chance of HPV-related cervical cancer?	No	280[77.3(72.7, 81.6)]	148[45.8(40.3, 51.4)]	P < 0.0001†
	Yes	82[22.7(18.5, 27.4)]	175[54.2(48.6, 59.7)]	
Do you know HPV can be transmitted through multiple sex partners?	No	287[79.3(74.7, 83.3)]	96[29.7(24.9, 35.1)]	p < 0.0001†
	Yes	75[20.7(16.7, 25.3)]	227[70.3(65.0, 75.2)]	
Do you know using condom/other birth control measures cannot prevent fully transmission of HPV?	No	285[78.7(74.1, 82.8)]	123[38.1(32.8, 43.6)]	p < 0.0001†
	Yes	77[21.3[17.2, 25.9)]	200[61.9(56.4, 67.2)]	
† Statistically Significant [p-Value < 0.05]				

## References

[1] Atkinson W Wolfe S Hamborsky J Centers for Disease Control and Prevention: Epidemiology and Prevention of Vaccine-Preventable Diseases. 12th ed. Washington DC: Public Health Foundation; 2012 http://www.cdc.gov/vaccines/pubs/pinkbook/hpv.html#hpv Accessed on 12 Jan 2014.

[2] Human papillomavirus (HPV) vaccine background paper. Geneva: World Health Organization, 2008. Accessed on 15th May, 2015Available on http://www.who.int/immunization/documents/HPVBGpaper_final_03_04_2009.pdf?ua=1

[3] Abreu AL Souza RP Gimenes F Consolaro ME A review of methods for detect human Papillomavirus infection. Virol J. 2012 ; https://doi.org/10.1186/1743-422X-9-262 10.1186/1743-422X-9-262 PMC3507852 23131123

[4] Burchell AN Tellier PP Hanley J Coutlee F Franco EL Human papillomavirus infections among couples in new sexual relationships. Epidemiology. 2010 ; https://doi.org/10.1097/EDE.0b013e3181c1e70b 10.1097/EDE.0b013e3181c1e70b PMC8313299 19907332

[5] Nour NM. Cervical cancer: a preventable death. Rev Obstet Gynecol. 2009 ; 2 (4): 240 - 4. PMid: PMCid: 20111660 PMC2812875

[6] Upreti D Regmi P Pant P Simkhada P Young people's knowledge, attitude, and behaviour on STI/HIV/AIDS in the context of Nepal: a systematic review. Kathmandu Univ Med J (KUMJ). 2009 ; 7 (28): 383 - 91. 20502079 10.3126/kumj.v7i4.2759

[7] Regmi PR Simkhada P van Teijlingen ER There are too many naked pictures found in papers and on the net": factors encouraging premarital sex among young people of nepal. Health Sci J. 2010 ; 4 (3): 169 - 81.

[8] Shah V Vyas S Singh A Shrivastava M Awareness and knowledge of cervical cancer and its prevention among the nursing staff of a tertiary health institute in Ahmedabad, Gujarat, India. Ecancermedicalscience. 2012 ; https://doi.org/10.3332/ecancer.2012.270 10.3332/ecancer.2012.270 PMC3437739 23008746

[9] Ferlay J Shin H Bray F Forman D Mathers C Parkin D Incidence/mortality data. GLOBOCAN 2008 v2.Cancer Incidence and Mortality Worldwide: IARC Cancer Base No. 10 [Internet]. Lyon, France: International Agency for Research on Cancer. 2010 . http://globocan.iarc.fr/factsheets/cancers/cervix.asp Accessed on 17 Feb 2014.

[10] Sherpa AT Clifford GM Vaccarella S Shrestha S Nygard M Karki BS et al Human papillomavirus infection in women with and without cervical cancer in Nepal. Cancer Causes Control. 2010 ; 21 (3): 323 - 30. https://doi.org/10.1007/s10552-009-9467-z 20217467 10.1007/s10552-009-9467-z

[11] Johnson DC Bhatta MP Smith JS Kempf MC Broker TR Vermund SH et al Assessment of high-risk human papillomavirus infections using clinician- and self-collected cervical sampling methods in rural women from far western Nepal. PLoS One. 2014 ; 9 (6): e101255 . https://doi.org/10.1371/journal.pone.0101255 10.1371/journal.pone.0101255 PMC4076302 24978811

[12] Human Papillomavirus and Related Cancers in Nepal, Fact Sheet 2014. ICO Information Centre on HPV and Cancer..

[13] Sankaranarayanan R Bhatla N Gravitt PE Basu P Esmy PO Ashrafunnessa KS et al Human papillomavirus infection and cervical cancer prevention in India, Bangladesh, Sri Lanka and Nepal. Vaccine. 2008 ; 26 Suppl 12 : M43 - 52. https://doi.org/10.1016/j.vaccine.2008.05.005 10.1016/j.vaccine.2008.05.005 18945413

[14] Dangal G Cervical Cancer and HPV Vaccine in Nepal: Issues and Challenges. Nepal J Obstet Gynaecol. 2012 ; 6 (2): 56 - 7. https://doi.org/10.3126/njog.v6i2.6761

[15] Joura EA Giuliano AR Iversen O-E Bouchard C Mao C Mehlsen J et al A 9-Valent HPV Vaccine against Infection and Intraepithelial Neoplasia in Women. New Engl J Med. 2015 ; 372 (8): 711 - 23. https://doi.org/10.1056/NEJMoa1405044 25693011 10.1056/NEJMoa1405044

[16] Singh Y Shah A Singh M Verma S Shrestha BM Vaidya P et al. Human papilloma virus vaccination in Nepal: an initial experience in Nepal. Asian Pac J Cancer Prev. 2010 ; 11 (3): 615 - 7. PMid: 21039025

[17] Gavi. Countries eligible for support. 2015.; http://www.gavi.org/support/apply/countries-eligible-for-support/ Accessed 15 May 2015.

[18] Perlman S Wamai RG Bain PA Welty T Welty E Ogembo JG Knowledge and awareness of HPV vaccine and acceptability to vaccinate in sub-Saharan Africa: a systematic review. PLoS One. 2014 ; https://doi.org/10.1371/journal.pone.0090912 10.1371/journal.pone.0090912 PMC3949716 24618636

[19] Nnodu O Erinosho L Jamda M Olaniyi O Adelaiye R Lawson L et al. Knowledge and attitudes towards cervical cancer and human papillomavirus: a Nigerian pilot study. Afr J Reprod Health. 2010 ; 14 (1): 95 - 108. PMid: 20695142

[20] Francis SA Nelson J Liverpool J Soogun S Mofammere N Thorpe RJ Jr Examining attitudes and knowledge about HPV and cervical cancer risk among female clinic attendees in Johannesburg, South Africa. Vaccine. 2010 ; https://doi.org/10.1016/j.vaccine.2010.08.090 10.1016/j.vaccine.2010.08.090 20887829

[21] Ayissi CA Wamai RG Oduwo GO Perlman S Welty E Welty T et al Awareness, acceptability and uptake of human papilloma virus vaccine among Cameroonian school-attending female adolescents. J Community Health. 2012 ; https://doi.org/10.1007/s10900-012-9554-z 10.1007/s10900-012-9554-z 22426995

[22] Chan ZC Chan TS Ng KK Wong ML A systematic review of literature about women's knowledge and attitudes toward human papillomavirus (HPV) vaccination. Public Health Nurs. 2012 ; https://doi.org/10.1111/j.1525-1446.2012.01022.x 10.1111/j.1525-1446.2012.01022.x 23078419

[23] Saha A Chaudhury AN Bhowmik P Chatterjee R. Awareness of cervical cancer among female students of premier colleges in Kolkata, India. Asian Pac J Cancer Prev. 2010 ; 11 (4): 1085 - 90. PMid: 21133629

[24] McCarey C Pirek D Tebeu PM Boulvain M Doh AS Petignat P Awareness of HPV and cervical cancer prevention among Cameroonian healthcare workers. BMC Womens Health. 2011 ; https://doi.org/10.1186/1472-6874-11-45 10.1186/1472-6874-11-45 PMC3219551 22008186

[25] Wamai RG Ayissi CA Oduwo GO Perlman S Welty E Manga S et al Assessing the effectiveness of a community-based sensitization strategy in creating awareness about HPV, cervical cancer and HPV vaccine among parents in North West Cameroon. J Community Health. 2012 ; https://doi.org/10.1007/s10900-012-9540-5 10.1007/s10900-012-9540-5 22302651

[26] Singh A Wong T Howlett RI Human papillomavirus vaccines: Why the time is right to implement immunization and surveillance programs in Canada. Can J Infect Dis Med Microbiol. 2008 ; 19 (4): 294 - 6. https://doi.org/10.1155/2008/813065 19436510 10.1155/2008/813065 PMC2604776

[27] Peat J Mellis C Williams K. Health science research: a handbook of quantitative methods. Sage; 2002.; . https://doi.org/10.4135/9781849209250

[28] van Teijlingen E Hundley V Pilot studies in family planning and reproductive health care. J Fam Plann Reprod Health Care. 2005 ; https://doi.org/10.1783/1471189054483735 10.1783/1471189054483735 16105287

[29] Joy T Sathian B Bhattarai C Chacko J. Awareness of cervix cancer risk factors in educated youth: a cross-sectional, questionnaire based survey in India, Nepal, and Sri Lanka. Asian Pac J Cancer Prev. 2011 ; 12 (7): 1707 - 12. PMid: 22126549

[30] Sathian B Sreedharan J Baboo SN Sharan K Abhilash ES Rajesh E Relevance of Sample Size Determination in Medical Research. Nepal J Epidemiol. 2010:; 1 (1): 4 - 10.

[31] Sathian B Methodological Rigors in Medical Journals from Developing Countries: An Appraisal of the Scenario in Asia. Nepal J Epidemiol. 2011 ; 1 (5): 141 - 43.

[32] WMA Declaration of Helsinki - Ethical Principles for Medical Research Involving Human Subjects [Internet]. 2013; http://www.wma.net/en/30publications/10policies/b3/index.html 19886379

[33] Sharma R Khadk B Gautam P Girl's Education in Nepal. Education Journalists Group, Nepal. 2003 ; http://ejg.org.np/report/Girl%27s_Education_in_Nepal.pdf Accessed on 01 Jan 2015.

[34] Cox T The current socio-economic status of untouchables in Nepal. Occas Pap Sociol Anthropol. 1994 ; 4 : 90 - 109.

[35] Walsh CD Gera A Shah M Sharma A Powell JE Wilson S Public knowledge and attitudes towards Human Papilloma Virus (HPV) vaccination. BMC Public Health. 2008 ; https://doi.org/10.1186/1471-2458-8-368 10.1186/1471-2458-8-368 PMC2579427 18947430

[36] Omer SB Orenstein WA Koplan JP Go big and go fast--vaccine refusal and disease eradication. N Engl J Med. 2013 ; https://doi.org/10.1056/NEJMp1300765 10.1056/NEJMp1300765 23574116

[37] Makwe CC Anorlu RI Knowledge of and attitude toward human papillomavirus infection and vaccines among female nurses at a tertiary hospital in Nigeria. Int J Womens Health. 2011 ; https://doi.org/10.2147/IJWH.S22792 10.2147/IJWH.S22792 PMC3181212 21976985

[38] Levine OS Bloom DE Cherian T de Quadros C Sow S Wecker J et al The future of immunisation policy, implementation, and financing. Lancet. 2011 ; https://doi.org/10.1016/S0140-6736(11) 10.1016/S0140-6736(11)60406-6 21664676

[39] Machingaidze S Wiysonge CS Hussey GD Strengthening the expanded programme on immunization in Africa: looking beyond 2015. PLoS Med. 2013 ; 10 (3): e1001405 . https://doi.org/10.1371/journal.pmed.1001405 10.1371/journal.pmed.1001405 PMC3601964 23526886

[40] Binagwaho A Wagner CM Gatera M Karema C Nutt CT Ngabo F Achieving high coverage in Rwanda's national human papillomavirus vaccination programme. Bull World Health Organ. 2012 ; https://doi.org/10.2471/BLT.11.097253 10.2471/BLT.11.097253 PMC3417784 22893746

[41] Moodley I Tathiah N Mubaiwa V Denny L. High uptake of Gardasil vaccine among 9 - 12-year-old schoolgirls participating in an HPV vaccination demonstration project in KwaZulu-Natal, South Africa. S Afr Med J. 2013 ; 103 (5): 318 - 21. https://doi.org/10.7196/SAMJ.6414 PMid: 23971122 10.7196/samj.6414

[42] Al-Meer FM Aseel MT Al-Khalaf J Al-Kuwari MG Ismail MF. Knowledge, attitude and practices regarding cervical cancer and screening among women visiting primary health care in Qatar. East Mediterr Health J. 2011 ; 17 (11): 855 - 61. PMid: 22276494 10.26719/2011.17.11.856

[43] Sait KH. Knowledge, attitudes, and practices regarding cervical cancer screening among physicians in the Western Region of Saudi Arabia. Saudi Med J. 2011 ; 32 (11): 1155 - 60. PMid: 22057604

[44] Lima EG de Lima DB Miranda CA de Sena Pereira VS de Azevedo JC de Araujo JM et al Knowledge about HPV and Screening of Cervical Cancer among Women from the Metropolitan Region of Natal, Brazil. ISRN Obstet Gynecol. 2013 ; https://doi.org/10.1155/2013/930479 10.1155/2013/930479 PMC3628759 23606981

[45] Johnson DC Bhatta MP Gurung S Aryal S Lhaki P Shrestha S. Knowledge and awareness of human papillomavirus (HPV), cervical cancer and HPV vaccine among women in two distinct Nepali communities. Asian Pac J Cancer Prev. 2014 ; 15 (19): 8287 - 93. https://doi.org/10.7314/APJCP.2014.15.19.8287 PMid: 25339019 10.7314/apjcp.2014.15.19.8287

[46] Arrossi S Maceira V Paolino M Sankaranarayanan R Acceptability and uptake of HPV vaccine in Argentina before its inclusion in the immunization program: a population-based survey. Vaccine. 2012 ; https://doi.org/10.1016/j.vaccine.2012.01.032 10.1016/j.vaccine.2012.01.032 22266289

[47] Winkler JL Wittet S Bartolini RM Creed-Kanashiro HM Lazcano-Ponce E Lewis-Bell K et al Determinants of human papillomavirus vaccine acceptability in Latin America and the Caribbean. Vaccine. 2008 ; https://doi.org/10.1016/j.vaccine.2008.05.027 10.1016/j.vaccine.2008.05.027 18945404

[48] Remes P Selestine V Changalucha J Ross DA Wight D de Sanjose S et al A qualitative study of HPV vaccine acceptability among health workers, teachers, parents, female pupils, and religious leaders in northwest Tanzania. Vaccine. 2012 ; https://doi.org/10.1016/j.vaccine.2012.06.025 10.1016/j.vaccine.2012.06.025 PMC3409375 22732428

[49] LaMontagne DS Barge S Le NT Mugisha E Penny ME Gandhi S et al Human papillomavirus vaccine delivery strategies that achieved high coverage in low- and middle-income countries. Bull World Health Organ. 2011 ; https://doi.org/10.2471/BLT.11.089862 10.2471/BLT.11.089862 PMC3209730 22084528

[50] Oh JK Lim MK Yun EH Lee EH Shin HR Awareness of and attitude towards human papillomavirus infection and vaccination for cervical cancer prevention among adult males and females in Korea: a nationwide interview survey. Vaccine. 2010 ; https://doi.org/10.1016/j.vaccine.2009.11.079 10.1016/j.vaccine.2009.11.079 20005860

[51] Wong LP Knowledge and attitudes about HPV infection, HPV vaccination, and cervical cancer among rural southeast Asian women. Int J Behav Med. 2011 ; 18 (2): 105 - 11. https://doi.org/10.1007/s12529-010-9104-y 20524163 10.1007/s12529-010-9104-y

[52] Poole DN Tracy JK Levitz L Rochas M Sangare K Yekta S et al A cross-sectional study to assess HPV knowledge and HPV vaccine acceptability in Mali. PLoS One. 2013 ; https://doi.org/10.1371/journal.pone.0056402 10.1371/journal.pone.0056402 PMC3576405 23431375

[53] Guimond ME Salman K Modesty matters: cultural sensitivity and cervical cancer prevention in muslim women in the United States. Nurs Womens Health. 2013 ; https://doi.org/10.1111/1751-486X.12034 10.1111/1751-486X.12034 23773193

[54] Pelucchi C Esposito S Galeone C Semino M Sabatini C Picciolli I et al Knowledge of human papillomavirus infection and its prevention among adolescents and parents in the greater Milan area, Northern Italy. BMC Public Health. 2010 ; https://doi.org/10.1186/1471-2458-10-378 10.1186/1471-2458-10-378 PMC2901377 20584324

[55] Madhivanan P Li T Srinivas V Marlow L Mukherjee S Krupp K Human papillomavirus vaccine acceptability among parents of adolescent girls: obstacles and challenges in Mysore, India. Prev Med. 2014 ; https://doi.org/10.1016/j.ypmed.2014.04.002 10.1016/j.ypmed.2014.04.002 24732716

[56] Marlow LAV Waller J Wardle J. Public awareness that HPV is a risk factor for cervical cancer. Br J Cancer. 2007 ; 97 (5): 691 - 4. https://doi.org/10.1038/sj.bjc.6603927 PMid: PMCid: 17687335 10.1038/sj.bjc.6603927 PMC2360359

[57] Anderson TA Schick V Herbenick D Dodge B Fortenberry JD A study of human papillomavirus on vaginally inserted sex toys, before and after cleaning, among women who have sex with women and men. Sex Transm Infect. 2014 ; https://doi.org/10.1136/sextrans-2014-051558 10.1136/sextrans-2014-051558 24739872

[58] Burchell AN Rodrigues A Moravan V Tellier PP Hanley J Coutlee F et al Determinants of prevalent human papillomavirus in recently formed heterosexual partnerships: a dyadic-level analysis. J Infect Dis. 2014 ; https://doi.org/10.1093/infdis/jiu200 10.1093/infdis/jiu200 PMC4192056 24683197

[59] Weber SK Schlagenhauf P Childhood vaccination associated adverse events by sex: a literature review. Travel Med Infect Dis. 2014 ; https://doi.org/10.1016/j.tmaid.2014.01.008 10.1016/j.tmaid.2014.01.008 24680600

[60] Antonsson A Cornford M Perry S Davis M Dunne MP Whiteman DC Prevalence and risk factors for oral HPV infection in young Australians. PLoS One. 2014 ; https://doi.org/10.1371/journal.pone.0091761 10.1371/journal.pone.0091761 PMC3956721 24637512

[61] Tang SY Liu ZH Li L Cai HL Wan YP. Awareness and knowledge about human papillomavirus among high school students in China. J Reprod Med. 2014 ; 59 (1-2): 44 - 50. PMid: 24597286

[62] Cox T The Badi: Prostitution as a social norm among an untouchable caste of West Nepal. Contrib Nepalese Stud. 1992 ; 19 (1): 51 - 71.

[63] Wamai RG Ayissi CA Oduwo GO Perlman S Welty E Welty T et al Awareness, knowledge and beliefs about HPV, cervical cancer and HPV vaccines among nurses in Cameroon: an exploratory study. Int J Nurs Stud. 2013 ; https://doi.org/10.1016/j.ijnurstu.2012.12.020 10.1016/j.ijnurstu.2012.12.020 23395482

[64] Mingo AM Panozzo CA DiAngi YT Smith JS Steenhoff AP Ramogola-Masire D et al Cervical cancer awareness and screening in Botswana. Int J Gynecol Cancer. 2012 ; https://doi.org/10.1097/IGC.0b013e318249470a 10.1097/IGC.0b013e318249470a PMC4437542 22367370

[65] Binagwaho A Wagner CM Nutt CT HPV vaccine in Rwanda: different disease, same double standard. Lancet. 2011 ; https://doi.org/10.1016/S0140-6736(11) 10.1016/S0140-6736(11)61837-0 22137840

[66] Ouedraogo N Muller O Jahn A Gerhardus A Human papillomavirus vaccination in Africa. Lancet. 2011 ; https://doi.org/10.1016/S0140-6736(11) 10.1016/S0140-6736(11)61164-1 21784261

[67] Larson HJ Brocard P Garnett G The India HPV-vaccine suspension. Lancet. 2010 ; https://doi.org/10.1016/S0140-6736(10) 10.1016/S0140-6736(10)60881-1 20728739

[68] Ilter E Celik A Haliloglu B Unlugedik E Midi A Gunduz T et al Women's knowledge of Pap smear test and human papillomavirus: acceptance of HPV vaccination to themselves and their daughters in an Islamic society. Int J Gynecol Cancer. 2010 ; https://doi.org/10.1111/IGC.0b013e3181dda2b9 10.1111/IGC.0b013e3181dda2b9 20683417

